# Quality and representativeness of research online with Yahoo! Crowdsourcing

**DOI:** 10.3389/fpsyg.2025.1588579

**Published:** 2025-08-21

**Authors:** Katie Seaborn, Satoshi Nakamura

**Affiliations:** ^1^Aspirational Computing Lab, Department of Industrial Engineering and Economics, Institute of Science Tokyo, Tokyo, Japan; ^2^Nakamura Laboratory, School of Interdisciplinary Mathematical Sciences, Meiji University, Nakano, Tokyo, Japan

**Keywords:** online sampling, sampling quality, participant characteristics, participant pool, recruitment, research quality, Japan

## Abstract

**Introduction:**

Conducting research online has become common in human participant research and notably in the field of human-computer interaction (HCI). Many researchers have used English-language and Western participant pool and recruitment platforms like Amazon Mechanical Turk and Prolific, with panel quality and representativeness known to vary greatly. Less is known about non-English, non-Western options. We consider Japan, a nation that produces a significant portion of HCI research. We report on an evaluation of the widely-used Yahoo! Crowdsourcing (YCS) recruitment platform.

**Methods:**

We evaluated 65 data sets comprising *N* = 60, 681 participants, primarily focusing on the 42 data sets with complete meta data from studies requiring earnest participation (*n* = 29, 081).

**Results:**

We found generally high completion (77.6%) and retention rates (70.1%). Notably, use of multimedia stimuli exhibited higher completion (97.7%) and retention (91.9%) rates. We also found that the “general” participant setting attracted middle-aged men.

**Discussion:**

We offer guidelines for best practice, such as online questionnaire design strategies to increase data quality and filtering to capture a more representative audience. We reveal the nature, power, and limitations of YCS for HCI and other fields conducting human participant research online.

## 1 Introduction

Online studies and questionnaires have become a common means of gathering user experience (UX) data and running panel surveys, user studies, and human participant-based experiments within human participant research generally (Bentley et al., 2020; Müller et al., 2014) and notably within the tech-centric field of human-computer interaction (HCI) (Gottfried, 2024; Landers and Behrend, 2015). A variety of online recruitment systems and participant pools have emerged. Some are multipurpose crowdsourcing platforms that are not necessarily geared toward research, such as the well-studied (Buhrmester et al., 2011; Bentley et al., 2020; Ross et al., 2010) Amazon Mechanical Turk (AMT). Others have been designed to gather insights and opinions through panels, like SurveyMonkey Collectors (Bentley et al., 2017, 2020). Still others have been specifically designed for research purposes, like Prolific and Qualtrics. What all have in common is their efficacy as a means for researchers to capture mass amounts of data from large and potentially representative samples within and beyond their base nation.

Online sampling and recruitment methods have an important role to play in enabling the replicability of research and fighting the replication crisis. Replication requires high quality data collection and analysis (Nature, 2022). Diversity in samples—from not relying on a single lab to demographic diversity, in the case of participant-based research—is a critical feature of replication quality (Nature, 2022). Online recruitment methods offer a flexible and quick means of gathering diverse samples, with the power to reach large and diverse samples in pursuit of generalizability (Müller et al., 2014). Notably, online methods can enable cross-cultural work. This is important for replication because of ongoing sample biases like WEIRD—samples originating from Wester, Educated, Industrial, Rich, and Democratic nations, which can bear striking differences to other populations (Henrich et al., 2010a,b)—and Anglocentrism (Levisen, 2019). Replication is needed outside of the original research team's base of operations to determine if a given effect is truly generalizable (Nature, 2022). An online panel can aid in such efforts, offering a cheaper and quicker option with similar quality and representativeness potentials when compared to traditional approaches like recruitment via market firms (Bentley et al., 2020). Recruiting online can also overcome common sampling biases, especially reliance on convenience sampling in the university or organization (Landers and Behrend, 2015). Finally, the greater reach of online sampling may help with previous limitations in replication work that exacerbate the crisis, notably by increasing the power of statistical analysis simply with the larger sample sizes that can be achieved online (Maxwell et al., 2015). Moreover, a greater number of individual studies can be run so as to capture how truly replicable an effect is (Maxwell et al., 2015; Nature, 2022). Altogether, online sampling has the potential to reduce variance in outcomes, rapidly and affordably replicate existing work, and involve a plethora of populations for greater generalization.

Still, online recruitment methods bear limitations and challenges. Participant pool quality can vary in terms of *performance* (Müller et al., 2014; Gottfried, 2024; Bentley et al., 2020; Landers and Behrend, 2015), including: **quit rates**, the number of participants who start the study but quit before completing it (Mittereder and West, 2021; Gottfried, 2024; Yamazaki et al., 2023b); **completion rates**, the number of participants who complete the study (Gottfried, 2024); and **retention rates** (or **data retention**), the number of participant data points that are preserved after checking the quality of the data, often a measure of data quality (Gottfried, 2024). Careless (Brühlmann et al., 2020; Curran, 2016; Muszyński, 2023) and **non-earnest** (Yamazaki et al., 2023c) responses—when participants rush through the study and make mistakes or otherwise provide poor data, including fake answers and gibberish—can hamper data quality. Performance is also tied to the *design of the survey*, such as the task demand and presentation of inputs (Yamazaki et al., 2023b,c), but also the checks that researchers (fail to) place. This includes **attention checks**, where irrelevant items are added to the questionnaire that can identify inattentive and non-earnest participants (Muszyński, 2023), and **technical checks** used to ensure that the necessary technical setup on the participant side is working, such as a sound-based password prompt to check audio-output settings. The *data quality evaluations* conducted by the research team (Gottfried, 2024) is also key. Strikingly, (Gottfried 2024) found that 55% of 3,298 psychology articles published in 2022 did not check data quality through such measures as response time (over or under), multiple submissions by the same person, self-report items as attention checks, and so on. Insufficient execution on any three of these matters can lead to false conclusion and poor knowledge production (Huang et al., 2015). Beyond quality, platforms also vary in terms of population *representativeness*: gender, age, education, lifestyle, language ability, cultural origin, and more (Bentley et al., 2020). The demographic composition of panels can also change over time (Ross et al., 2010) and may need to be periodically re-assessed.

As Landers and Behrend (2015) and Roulin (2015) argue, choice of an online panel and which specific recruitment platform should be a considered and informed decision. This has inspired several efforts to assess the quality of specific platforms (Bentley et al., 2020; Roulin, 2015; Müller et al., 2014). Nevertheless, this important trajectory of metascience has thus far been subject to the complex and wide-scale WEIRD (Henrich et al., 2010a,b) and Anglocentric (Levisen, 2019) biases. The human participant research, including in HCI fields (Linxen et al., 2021; Seaborn et al., 2023a; Seaborn and Chang, 2024), that we rely on for general knowledge is largely based on sampling from English-centric Western, Educated, Industrialized, Rich, and Democratic nations. While some participant pools offer access to samples from several nations, the level of access may be low. In our experience, there are relatively few Japanese participants on AMT and Prolific, for instance. Yahoo! Crowdsourcing JAPAN (YCS), the market dominant option, reportedly has 85 million users as of November 2024^1^. Very little work so far has considered the actual reach or quality of the sociolinguistically diverse samples on these platforms, with most work so far focusing on English and Western (typically US-based) samples (Bentley et al., 2020; Gottfried, 2024). Moreover, platforms created by and for certain nations may have strengths and perks other platforms cannot offer. For example, YCS requires one account per person with ID verification and phone tethering.

As a first step toward addressing this “WEIRD” gap, we consider the case of Japan, an “EIRD” nation with a population that largely speaks Japanese and is a major non-Western contributor to HCI research (Linxen et al., 2021). We focus on YCS as the leading platform with the greatest uptake across Japan. YCS was originally created by Yahoo! JAPAN, a joint endeavor between Yahoo! and the Japanese electronics company SoftBank. YCS is now under the purview of LY Corporation (LYC), a result of the merger between LINE Corporation and Yahoo! JAPAN. YCS crowdsourcers earn compensation as points through the cashless payment service PayPay. Accounts are tied to a single phone number^2^, and re-registering after account deletion takes 6 months^3^. As yet, the data quality of YCS remains uncharted. Yet, HCI researchers working in Japan or with collaborators in Japan may use the platform and be asked (as we have) by reviewers and readers to justify its quality (and rightly so).

In this study, we aimed to reveal the data quality and representativeness of YCS as flagship recruitment platform and participant pool for human participant research in Japan. To this end, we asked several research questions (RQs). **RQ1**: *What data quality does YCS offer in terms of completion rates and data retention?*, **RQ2**: *How representative is the data in terms of population gender and age?*, and **RQ3**: *What features of the study design improve data quality?* We analyzed 65 data sets covering a five-year span (2020–2025) that comprise *N* = 60, 681 participants who partook in a range of experimental and non-experimental tasks. We offer the following contributions:

- **Research**: We demonstrate, with studies of varying scales that cover a breadth of topics, the benefits and weaknesses of the data collected through YCS. These empirical findings may help explain the results of researchto a general audience.
- **Methodological**: We reveal what settings on YCS and what features of the study design can be manipulated to ensure higher data quality and representativeness. We bring in extant work to offer standard guidance contextualized for YCS.
- **Practical**: We provide empirical findings on the quality of YCS for the research community. This work can be shared with those outside of Japan and notably cited in reports and responses to reviews in answer to questions about the nature and quality of YCS, saving time and space for Japan-based researchers participating in the international community of practice.

In pursuit of open science, we also offer our open data set, which includes the metadata on the data sets used in our analysis: https://bit.ly/ycjdataquality. We hope that this work can help Japan-based researchers make informed choices about use of the platform, justify its use, and perhaps contribute to future data quality checks. Notably, these data sets may be used for future comparisons with other platforms in Japan or abroad. Such comparisons may help researchers make sense of the relative data quality for the online studies we conduct and could provide empirical evidence of needed changes in specific platforms to improve quality based on international standardization.

## 2 Materials and methods

We carried out descriptive and comparative analyses of the YCS data sets gathered from two Japan-based human participant research labs and published research. Tables 1, 2 provide an overview of the data sets and sources. Our variables of analysis were guided by our RQs and related literature in HCI (Bentley et al., 2020, 2017) and more broadly (Müller et al., 2014; Gottfried, 2024). Notably, we considered the role of *mediated experiences*—sound and video, interactive websites—in completion and retention rates, as these may be more demanding and naturally exclude lower-performing and less interested participants. Our comparative analyses were based in fundamental differences between the data sets (refer to Section 3.1). Our goal was to thickly describe and tease out the nature of YCS data production and factors related to quality.

**Table 1:** Overview of data sets (part one of two).

**ID**	**Topic**	**Stim**.	**Checks**		**Quit**		**Completion**	**Retention**	**Source**
			**A**.	**T**.	**Count**	**%**			
1	Game experience	Text			7	12.3%	0.98	0.86	(Seaborn et al., 2024b)
8	Voice experience	Sound	○	○	0	0.0%	1.00	1.00	(Mandai et al., 2025)
9	Game character impressions	Sound	○	○	0	0.0%	1.00	1.00	
10	Voice experience	Sound	○	○	0	0.0%	0.98	0.98	
13	Voice experience	Sound	○	○	5	8.9%	1.00	0.91	(Mandai et al., 2025)
2	Voice experience	Sound	○		14	13.0%	1.00	0.87	(Seaborn et al., 2023b, 2025)
3	Voice experience	Sound	○	○	3	2.9%	1.00	0.97	
4	Voice experience	Sound	○	○	6	3.8%	1.00	0.96	
5	Robot impressions	Text + Images			8	4.9%	0.99	0.94	
7	Voice experience	Sound	○	○	9	5.3%	0.98	0.93	(Seaborn et al., 2023c)
11	Game character impressions	Sound	○	○	6	3.8%	0.99	0.96	
14	Voice experience	Sound	○	○	0	0.0%	1.00	1.00	
15	AI impressions	Video (no sound)	○	○	16	7.4%	1.00	0.93	(Fujii et al., 2024)
16	AI impressions	Video + Sound	○	○	11	5.1%	1.00	0.95	(Fujii et al., 2025)
6	AI impressions	Text			22	7.9%	1.00	0.92	
12	Game character impressions	Sound + Images	○	○	40	13.2%	1.00	0.86	
17	Chatbot/CUI experience	Text			22	7.9%	0.78	0.71	(Seaborn et al., 2024a)
59	Aquarium & zoo				65	6.1%	0.99	0.93	(Nakagawa and Nakamura, 2023)
52	Browsing	Interactive	○	○	0	0.0%	0.56	0.56	
31	Calculation	Interactive	○	○	117	10.5%	0.79	0.70	
50	Card task + pointing	Interactive	○	○	151	24.9%	0.76	0.57	
49	Card task + progress bar	Interactive	○	○	210	43.3%	0.69	0.39	
18	Comic				N/A	N/A	0.75	N/A	
37	Comic				749	42.8%	0.88	0.50	
58	Cooking				54	0.99	0.94	93.8%	(Matsuda and Nakamura, 2024)
60	Desk work in COVID-19		○		N/A	N/A	0.97	N/A	
30	Drawing				N/A	N/A	0.76	N/A	
36	Driving				757	43.1%	0.98	0.56	(Yamazaki et al., 2023a)
32	Driving difficulty		○		0	0.0%	0.94	0.94	(Nakagawa et al., 2023)
19	Fashion				N/A	N/A	0.96	N/A	
56	Font impression	Image	○		N/A	N/A	1.00	N/A	
54	Handwriting + selection	Image	○		N/A	N/A	0.92	N/A	
26	Makeup				N/A	N/A	0.77	N/A	
29	Makeup				N/A	N/A	0.98	N/A	(Kajita and Nakamura, 2021)
33	Memory	Text	○	○	N/A	N/A	0.87	N/A	

A., Attention; Stim., Stimulus; T., Technical.

**Table 2:** Overview of data sets (part two of two).

**ID**	**Topic**	**Stim**.	**Checks**		**Quit**		**Completion**	**Retention**	**Source**
			**A**.	**T**.	**Count**	**%**			
34	Memory	Text	○	○	N/A	N/A	0.80	N/A	
57	Memory	Text + Images	○	○	N/A	N/A	0.94	N/A	
53	Sports spoiler				N/A	N/A	0.97	N/A	
28	Oshigatari				N/A	N/A	0.82	N/A	(Funazaki and Nakamura, 2022)
46	Progress bar (browsing)	Interactive	○	○	75	11.1%	0.72	0.64	
21	Selection bias	Interactive	○	○	487	32.8%	0.62	0.42	(Yokoyama et al., 2021)
22	Selection bias	Interactive	○	○	69	6.4%	0.61	0.57	
23	Selection bias	Interactive	○	○	41	3.9%	0.63	0.60	
24	Selection bias	Interactive	○	○	38	3.7%	0.34	0.33	
25	Selection bias	Interactive	○	○	89	4.3%	0.51	0.49	
41	Selection bias - color	Interactive	○	○	35	3.4%	0.90	0.87	(Sekiguchi et al., 2023)
42	Selection bias - delay	Interactive	○	○	40	3.8%	0.91	0.88	(Kinoshita et al., 2023)
43	Selection bias - delay	Interactive	○	○	24	2.3%	0.90	0.88	(Kinoshita et al., 2023)
44	Selection bias - delay	Interactive	○	○	59	2.9%	0.74	0.72	
39	Selection bias - font	Interactive	○	○	211	17.4%	0.95	0.78	(Ueki et al., 2023)
40	Selection bias - font	Interactive	○	○	N/A	N/A	0.94	N/A	(Ueki et al., 2023)
47	Selection bias - image order	Interactive	○	○	123	5.8%	0.82	0.77	
48	Selection bias - image order	Interactive	○	○	181	8.3%	0.83	0.76	
45	Selection bias - pre	Interactive	○	○	108	5.1%	0.74	0.71	
51	Selection bias - progressive	Interactive	○	○	71	6.6%	0.41	0.38	
38	Smartphone		○		1131	36.1%	0.89	0.57	(Yamazaki et al., 2023b)
35	Telework				344	23.8%	1.00	0.76	(Yamazaki et al., 2023a)
55	Time estimation	Interactive	○		N/A	N/A	0.50	N/A	
20	Waiting				N/A	N/A	0.96	N/A	
27	Word of mouth				N/A	N/A	1.00	N/A	
61	Conjunction test	Text	○		11	5.2%	1.04	0.98	
62	Accounts	Text	○		407	11.0%	N/A	N/A	
63	Online shop	Interactive	○	○	39	4.9%	0.99	0.94	
64	Voice experience	Sound	○	○	11	3.4%	0.99	0.96	
65	Accounts	Text	○		0	0.0%	0.68	0.68	

A., Attention; Stim., Stimulus; T., Technical.

Several approaches were used for data analysis. Descriptive statistics, including counts and percentages, as well as mean (M), standard deviation (SD), median (MD), and interquartile range (IQR) were generated. Comparative analyses involved visualization through bar charts and stacked bar charts. Quit rate was determined based on the metadata offered by YCS: a ratio of the *sum* of **timeouts**, or how many participants did not finish the study in time, and **cancellations**, or how many participants canceled the study after starting, over **access rates**, or how many participants started the study. In some cases, timeouts were manually included or participants emailed us to be manually excluded; additions and subtractions were made to account for these. Data quality measures for retention were coded by the first author using the codebook developed by Gottfried (2024). Specifically, this included: *response time*, or the time spent completing the survey; *self-reports* of study engagement, knowledge, or answer validity; *multiple submissions* controls for individual respondents; *cross-check*, or comparing to data from a second source; *consistency of answers* to assess whether the answers are logically or theoretically coherent; *control items*, or use of implausible, rare, or nonsensical items as attention checks; *outliers*, or extraordinarily scores; *missing rates*, or the proportion of unanswered questions; *variability* (or a lack thereof) in response patterns; *open answer* quality checks; and *other*, if none of the above apply.

Data analysis was complicated by differences in metadata records and instrumentation. For example, quit rates were not always provided, and age range categories varied across the data sets. Importantly, we did not include five studies from Lab B in our removal and analysis, as these studies intentionally included data from non-earnest participants in order to analyze the differences between earnest and non-earnest participants, which would unduly influence the retention rate. We also excluded these studies plus one from Lab A and a further 18 from Lab B in certain analyses, when the necessary data was not available (N/A) for analysis. We do our best to analyze all data while signposting these gaps and complexities.

## 3 Results

A total of 65 data sets were included, amounting to a recruitment pool of 60,681 (from a planned total of 58,777, a 3.2% surplus). Excluding one data set from Lab A still under analysis at the time of writing, there were analyzable data for 48,139 out of 57,127 participants. All studies were conducted between 1/27/2020 and 2/26/2025, a span of 5 years and 1 month.

### 3.1 Nature of the data sets and studies

The data sets were sourced from two Japan-based HCI labs. Lab A has predominantly carried out **multimedia** research involving voice clips, video, images, and interactives, like working prototypes of online stores accessed outside of the questionnaire (*n* = 22). Lab B has mostly conducted text-based online **surveys** and **experiments** involving off-survey engagements and custom platforms (*n* = 43). In terms of **platform of delivery**, Lab A mostly employed SurveyMonkey (18, 82%), while also using jsPsych once and Google Forms thrice. Lab B mostly used original internal platforms (32, 73%) or the survey features on YCS itself (10, 22%) and Google Forms (2, 5%).

Both labs employed **attention checks** (47, 72.3%), such as requiring a participant to select an unintuitive Likert scale option (e.g., Disagree) from a special opening question (e.g., “We are concerned about data quality, so please select Disagree to continue.”). Both labs also employed **technical checks** (36, 55.4%), like a short embedded video that speaks out a number for the participant to input, thereby confirming access to audio output, e.g., speakers or headphones, if required for the study.

Research **topic** varied by lab. Lab A targeted voice experience (12, 55%), game experience and impressions (5, 23%), and LLM and AI impressions (4, 18%). Subsequently, Lab A uniquely employed **sound** (12, 55%) and **video** (2, 9%) stimuli. Lab B focused on selection bias (7, 16%), with the rest representing a range topics, such as memory and cognition, telework, fandom, driving tasks, fashion and makeup, smartphones and UI, and writing and drawing.

### 3.2 Completion rates and data retention (RQ1)

**Figure 1:**
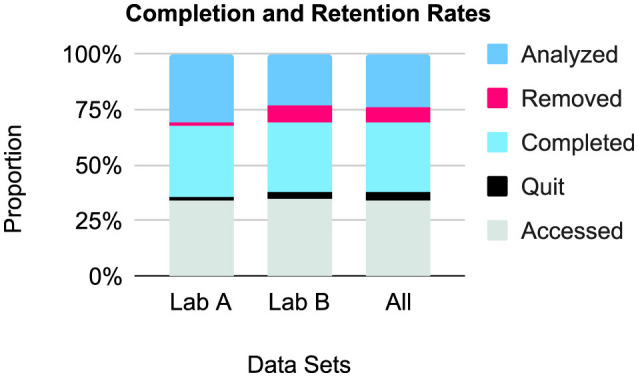
Relative completion and retention rates (*N* = 42), by lab and combined. Data from the non-earnest participation studies (*n* = 5) and data sets with missing data (*n* = 18) were not included.

Relative completion and retention rates, comprising accesses, quit numbers, completed entries, removals, and analyzed data, are presented in Figure 1. Note that these results only include the *N* = 42 (with *n* = 21 from each lab) that had all of the listed metadata and were not studies of non-earnest participation.

There was an average **quit rate** of 9.7% (*n* = 3, 108; data unavailable for 17 data sets and not including the *n* = 5 non-earnest participation studies). Lab B had higher quit rates. This may be explained by the generally larger sample sizes pursued in comparison to Lab A, which take longer to achieve and increase the likelihood of dropouts. Seven studies had a 0% quit rate: five in Lab A and two in Lab B. The largest quit sample was 1,131 (36.1%) and the highest quit rate was 43.3% (*n* = 210), both in Lab B.

The **completion rate**, was 77.6%, after making the above exclusions. Lab A included 3,622 participants (*M* = 172.5, *SD* = 153.8, *MD* = 153, *IQR* = 153) for a 97.7% completion rate, and Lab B included 18,947 participants (*M* = 902.2, *SD* = 593.0, *MD* = 881, *IQR* = 592) for a 74.7% completion rate. Lab B apparently had lower completion rates overall, which can also be explained by the sampling procedure.

**Figure 2:**
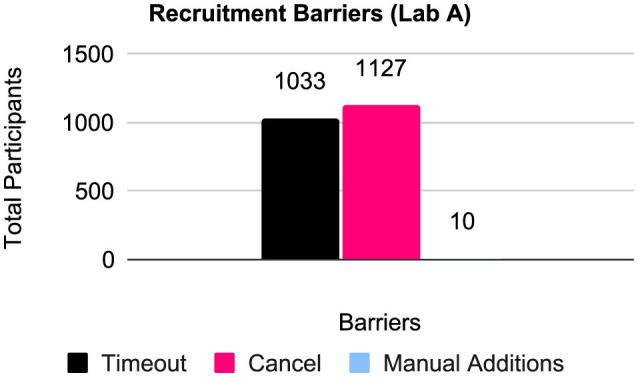
Recruitment barriers for Lab A data sets (*n* = 21).

Lab A also gathered **recruitment barriers** as provided by YCS in recruitment metadata. This metadata covered 7,627 potential participants across 21 data sets (Figure 2). These included: timeouts, where a potential participant signed up for the study but did not complete it on time in YCS (*n* = 1, 033, *M* = 47.0, *SD* = 208.2); cancellations, where a potential participant signed up but then changed their mind and canceled on YCS (*n* = 1, 127, *M* = 51.2, *SD* = 187.8); and manual additions, where the research team was contacted by participants separately, who provided evidence of participation but could not do so officially on YCS, typically due to technical issues like Internet outages (*n* = 10, *M* = 0.6, *SD* = 1.5).

On **removals**, we again excluded the non-earnest data sets (*n* = 5) and data sets missing data. Overall, 6,539 **removals** were made (22.5% of completed entries; *M* = 155.7, *SD* = 226.8, *MD* = 55.5, *IQR* = 203.75). Removal rates seemed to be higher for Lab B, which can be traced back to the quit and completion rates. 13 had a 0% removal rate: 11 in Lab A and two in Lab B. The largest was 979 (49%).

A total of 42,297 participants were included for **analysis**; here, we included data sets missing metadata but excluded the *n* = 5 non-earnest data sets and the data set from Lab A still being processed. If we exclude the data sets with missing data (26 from Lab B), then 22,569 out of 32,190 were included. This indicates a **retention rate** of 70.1% across labs. Breaking it down, Lab A included 3,622 of 3,940 (retention rate of 91.9%) and Lab B included 18,947 of 28,250 (retention rate of 67.1%). This will be evaluated further through study comparisons by lab in 3.4.

### 3.3 Representation by gender and age group (RQ2)

#### 3.3.1 Gender

**Figure 3:**
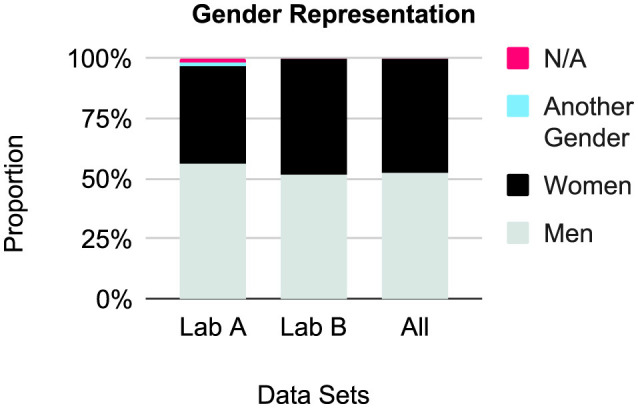
Relative gender representation, by lab and combined.

Results are presented in Figure 3, Table 3. There were few gender-diverse participants (*n* = 135, *M* = 2.1, *SD* = 6.8, *min* = 0, *max* = 35), and 112 people (*M* = 1.8, *SD* = 5.6, *min* = 0, *max* = 34) did not respond. The proportion of men and women for Lab A was 0.71 (men: 2,059, women: 1,460) and for Lab B was 0.92 (men: 23,491, women: 21,623). Overall, Lab A gathered more men than Lab B.

**Table 3:** Demographics by gender across data sets and by lab.

	**Men**	**Women**	**Another gender**	**N/A**
Lab A	2,059	1,460	47	78
Lab B	23,491	21,623	88	34
All	25,550	23,083	135	112

#### 3.3.2 Age group

**Figure 4:**
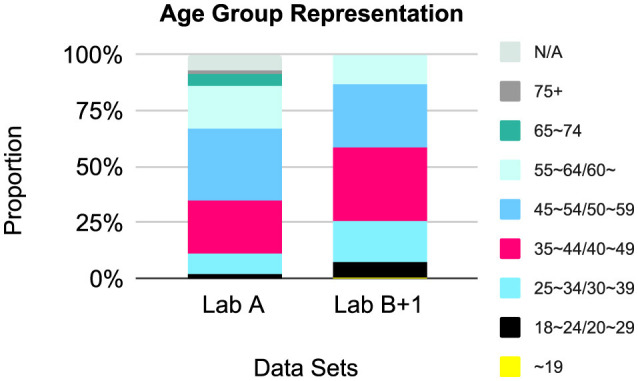
Relative age group representation, by lab and combined.

Representation by **age group** is presented in Figure 4 and Table 4. Each lab captured age ranges in a different way, with the exception of one study, which was included in the Lab B analysis as +1. As such, we urge caution when interpreting the figure, where we have combined similar age categories. Face valid assessments of the ranges indicate that there is an over-representation of people aged 35~59, i.e., middle-age, across data sets.

**Table 4:** Demographics by age across data sets and by lab.

**Age group**	**18–24**	**25–34**	**35–44**	**45–54**	**55–64**	**65–74**	**75+**	**N/A**
Lab A	63	351	850	1,179	704	188	53	256
**Age group**	~**19**	**20–29**	**30–39**	**40–49**	**50–59**	**60**~		**N/A**
Lab B+1	377	3,246	8,946	16,311	14,028	6,492		11

### 3.4 Data quality and study design (RQ3)

We considered 42 data sets where removals were made and a reason was reported, excluding the non-earnest participation studies (*n* = 5) and the one from Lab A under processing. Lab A reported on all ten, while Lab B did not report on six of 32. Of these, **data quality evaluation methods**, i.e., the reasons for data removal as per (Gottfried, 2024), included: control items, e.g., attention checks (28, 32%), response time (16, 18%), answer consistency (12, 14%), open answer, e.g., gibberish (13, 16%), missing rates (8, 9%), other (5, 6%), self-report (easy to answer but fail; 4, 5%), and one each of multiple submissions and variability, e.g., acquiescence bias. There were no cross-checks. Quit rates were higher for Lab B (10.2%) compared to Lab A (5.8%). Of the four Lab A data sets with a 0% quit rate, only one removal was made. All involved sound or video media and signposted this requirement before the informed consent stage. For Lab B, one 0% quit rate data set involved *n* = 130 removals (44% of participants recruited) and the other *n* = 157 (6%). In short, the data sets from each lab feature differences in sample size, sampling procedure, and other variables. Notably, Lab A relied on multimedia, while Lab B rarely used such stimuli. Determining whether this played a role in quit and removal rates should be a focus of future work.

## 4 Discussion

Our analysis of 65 data sets from two HCI labs utilizing YCS revealed generally high completion (77.6%) and retention rates (70.1%), with data quality depending on the stimuli and task demand (**RQ1**). Even so, representativeness (**RQ2**) and data quality varied (**RQ3**). Notably, attention checks and technical tests seemed to produce higher data retention for analysis. On representation, few gender-diverse people were recruited, with the default “general population” setting tending to engage middle-aged men. We encourage the reader to peruse the open data set (https://bit.ly/ycjdataquality).

How does YCS fare on the global stage? Completion rates varied by lab, from 74.7%~97.1%. Those found for HCI-related work fall within this range: 92% and 97% for SurveyMonkey Collectors and 92% for AMT (Bentley et al., 2020). This is good news for researchers aiming to conduct cross-cultural work, whether to assess generalization or conduct replication studies (Nature, 2022; Maxwell et al., 2015), which some within HCI (Ono et al., 2023; Seaborn et al., 2024b,a) and elsewhere (Youn et al., 2019) have already started to do. Additionally, the patterns we found match those in non-Japanese and WEIRD recruitment platforms. In Bentley et al. (2020), for instance, samples varied in terms of gender and age group range, with similar over-representation of men for AMT, but not SurveyMonkey Collectors. However, while some over-representation in the middle-age category of 45~54 was found, a trend toward younger age groups was more common for AMT. In short, special measures (discussed in 4.1) are needed to achieve representation and confidence when aiming to generalize or replicate work.

We also discovered a difference in the number of men and women recruited by lab. The relative distribution of men and women can vary by platform, with Bentley et al. (2020) showing lower rates of women for Amazon Mechanical Turk (33%~47%) but closer relative numbers to men in SurveyMonkey. In our case, the difference traces back to YCS settings. YCS offers a *general population* sample procedure, a well as targeted procedures, but only for *men* and *women*, therefore excluding unknown numbers of gender-diverse people (Spiel et al., 2019). Each lab addressed this issue in a different way. Lab A, aiming to capture gender-diverse participants, first recruited with the general default setting. Since the sample was nearly always overwhelmingly middle-aged men, they typically followed up with a smaller recruitment for women. However, sample size must be input at intervals of 50, leading to uneven samples. Lab B carried out parallel recruitment of men and women, ensuring nearly equal sample sizes for these gender identities but often leading to the exclusion of gender-diverse participants. Both options have drawbacks. Ideally, YCS should update their sample filter procedure for rigor and diversity.

The nature of the recruiting procedure on YCS led us to consider the intersections of gender and age. The use of the default sampling setting in YCS by Lab A in contrast to the targeted majority gender settings by Lab B revealed that general YCS sampling skews toward men. Moreover, the age results across data sets for both labs indicate a middle-aged bias. We believe this is linked to the Japanese work context. In Japan, salaried men, or the “salaryman,” are over-represented in the commute (Chowdhury and McFarlane, 2021), where smartphone use is prevalent. A review of the gender and age statistics provided by LYC in November 2024 (refer to text footnote 1) reflects this demographical intersection. On age, LYC only offers pie charts. According to these, the 40~60 age groups take up roughly two-thirds of smartphone and PC users. By gender, they report slightly more women (52.1%) using smartphones but more men using PCs (63.1%). Although the data sets did not include device information, this seems to reflect the sampling bias we found, too. While representative of the current situation in Japan, researchers who aim to replicate studies, especially where gender is a known factor, will need to identify and account for such sampling biases. As Borsa et al. (2012) recommend, conducting a pilot test with a researcher within the targeted cultural context may be ideal.

A potential element that may increase data quality is the use of multimedia stimuli. While we did not experimentally control for this possibility, our exploratory analyses of Lab A suggest that sound or video may be a variable in completion (97.7%) and retention (91.9%) rates (compared to the respective general rates of 77.6% and 70.1% for Lab B). Indeed, merely signposting and utilizing manipulation checks for multimedia—sound, video, and interactive experiences like use of online shopping websites in the study—may attract serious participants (and detract others). We stress that this finding is preliminary and exploratory. In future work, an opportunistic researcher could plan a longitudinal meta-study to experimentally control the collection of data in multimedia and non-multimedia studies when running basic research. Otherwise, collection of more data sets will be needed to determine whether other features of the research, such as sample size or topic, were more influential than the presence of multimedia.

### 4.1 Strategies for quality and representation with YCS

YCS offers a robust online recruitment platform. Nevertheless, our results indicate several shortcomings and potential tweaks to the settings and recruitment procedures to increase rigor and representation. Here, we offer the following suggestions for best practice when using YCS.

- **Check the quality**: Gottfried (2024) raised the alarm about lack of quality checks on the researcher's side. All online methods should be assessed for quality, every time. Our findings indicate that quality checks are necessary for YCS even when completion rates are high. Given our limited scope here, we recommend that interested parties reference the methods gathered by Gottfried (2024).
- **Use attention and technical checks**: A notable finding that nevertheless requires experimental verification was the apparent superior performance of studies involving multimedia, namely sound and/or video. Use of multimedia stimuli required technical and manipulation checks, which could help explain the increase in data retention. While a staple for particular domains like voice UX (Seaborn et al., 2025), these non-textual and dynamic checks may be repurposed for any study. Even so, use of sound or video may needlessly exclude valid participants, such as people on the train without headphones and people who are deaf or hard-of-hearing. Alternatives may need to be provided, which future work can explore.
- **Check for earnest/careless participation**: Attention and technical checks may not be enough, especially when used at the start of a study (Yamazaki et al., 2023c; Brühlmann et al., 2020; Curran, 2016). Subsequent checks may enable researchers to determine whether participants were fully engaged over the course of the study. This may require special development or customization of the platform. Participants can be given a unique code at the end of the study alongside a code after the key task, one that reflects whether the task was engaged with properly. The participant then inputs both to receive compensation. However, the researchers can later judge performance quality based on the second code.
- **Use multiple recruitment calls for the same study**: Our findings indicated a gender bias that is tricky to address. The default, general recruitment setting captures gender-diverse people, but men are over-represented. There is currently no option on YCS to target genders other than men or women. For best practice (Spiel et al., 2019), we recommend multiple calls of either (a) general and women or (b) general, men, and women, with planned sample sizes set highest for general and higher for women, i.e., 200 general, 100 women, 50 men.

### 4.2 Limitations

Our work bears several limitations. The number of data sets we evaluated was relatively small, and originated from only two research groups. Each group used different data collection methods for similar data, notably in terms of response formats for gender and age alongside analysis approaches. Sample size also varied greatly among the two labs. The research itself was diverse in format and time expectancy. For example, some studies were one-off questionnaires, while others were multi-phased experiences. This may have affected completion rates. While limitations, these differences also allowed us to provide a holistic view of YCS for common types of research, in and outside of HCI. Future work can contribute to the open data set (https://bit.ly/ycjdataquality) for subsequent analyses on more diverse research purposes and designs. This may allow for finer-grained analyses of demographics (if theoretically motivated) and features of the research design (such as use of specific kinds of media).

## 5 Conclusion

We evaluated a suite of data sets across a diverse range of HCI studies that employed YCS. This work offers empirical benchmarking to inform validity and reproducibility with online recruitment methods. We found generally high completion and retention rates, but some sampling biases. Future work should explore the role of multimedia and perhaps develop a general media-based attention check for use in any study. Future work should also consider the role of device—smartphone or PC—as an explanatory variable for dropouts. While providing insights on platform selection and recruitment procedures in pursuit of generalization and replication, our results should be replicated, too. Since our focus was on HCI, future work should evaluate samples gathered through YCS for other kinds of human participant research. Recruitment platforms that are, like YCS, only available within a certain country should also be assessed. Together, we can establish the degree to which online human participant research can be representative, rigorous, and replicable.

## Data Availability

The original data can be found here: https://bit.ly/ycjdataquality.

## Footnotes

^1^ https://www.lycbiz.com/sites/default/files/media/jp/download/LY_Corporation_MediaGuide.pdf

^2^ https://support.yahoo-net.jp/PccLogin/s/article/H000010762

^3^ https://paypay.ne.jp/help/c0011/
